# Supplementary material for: Glycolipid transfer protein modulates vesicular trafficking from the endoplasmic reticulum in HeLa cells
Source: J Biol Chem. 2026 Mar 17;302(5):111378. doi: 10.1016/j.jbc.2026.111378 (PMC13090591; doi:10.1016/j.jbc.2026.111378)
Supplement: Supplementary Material [file mmc1.pdf]

Supporting information

Glycolipid transfer protein modulates vesicular trafficking from the endoplasmic reticulum in HeLa cells

Henrik Nurmi, Linda Englund, Alina Henriksson, Max Lönnfors, Peter Mattjus*

Biochemistry & Cell Biology, Faculty of Science and Engineering, Åbo Akademi University, Artillerigatan 6A, III, 20520 Turku, Finland

* For correspondence: Peter Mattjus, peter.mattjus@abo.fi

Supplementary Figures

Figure S1. 3D surface plots of HeLa cells and quantification of fluorescence intensity for Sec23A.

Figure S2. 3D surface plots of HeLa cells and quantification of fluorescence intensity for UGCG, FAPP2 and CERT.

Figure S3. Anti-GlcCer and anti-GalCer antibody verification.

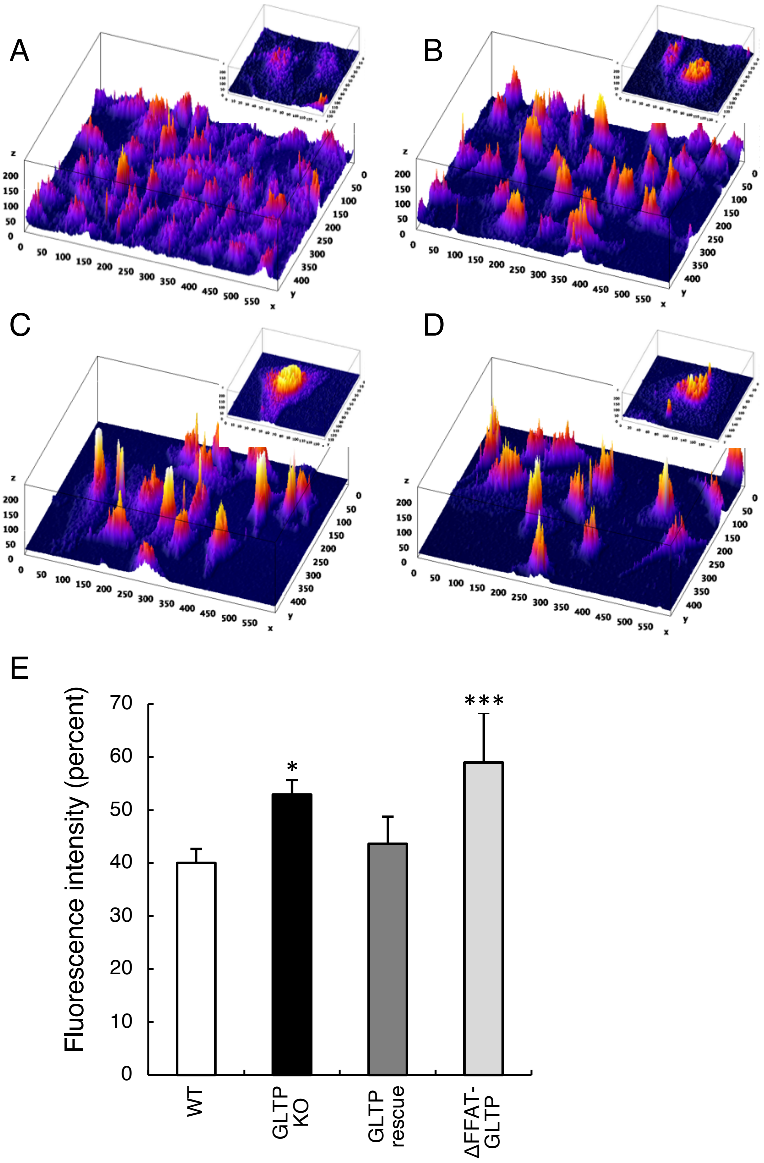

Figure S1. **3D surface plots of HeLa cells and quantification of fluorescence intensity for Sec23A**. We analysed the intensity of the fluorescence in HeLa cells with the ImageJ software using the 3D interactive surface plot to further analyze the changes in the intracellular localization of Sec23A as a function of GLTP knockout. We used ImageJ software to measure fluorescence intensity within specific regions of interest (ROI), including the nuclear, perinuclear, and ERES regions, and compared these values to the overall intensity of the entire cell body. (A) Surface plot of the fluorescence intensity for the expression of Sec23A in WT HeLa cells and (B) the expression of Sec23A in GLTP KO HeLa cells. (C) & (D) shows the fluorescence intensity of Sec23A in GLTP rescue HeLa cells and ΔFFAT-GLTP expressing HeLa cells respectively. (E) The fluorescence intensity was measured in defined ROIs corresponding to ER exit sites and the nuclear and perinuclear area and compared to the fluorescence intensity of the entire cell body. Data are presented as percent of intensity for the ROI ± SEM from at least 15 cells across independent experiments. The significance in difference between the WT group was tested using a Student's t test: *$p < 0.05$, ***$p < 0.001$. The subpanels are higher magnifications (2x) of representative cells.

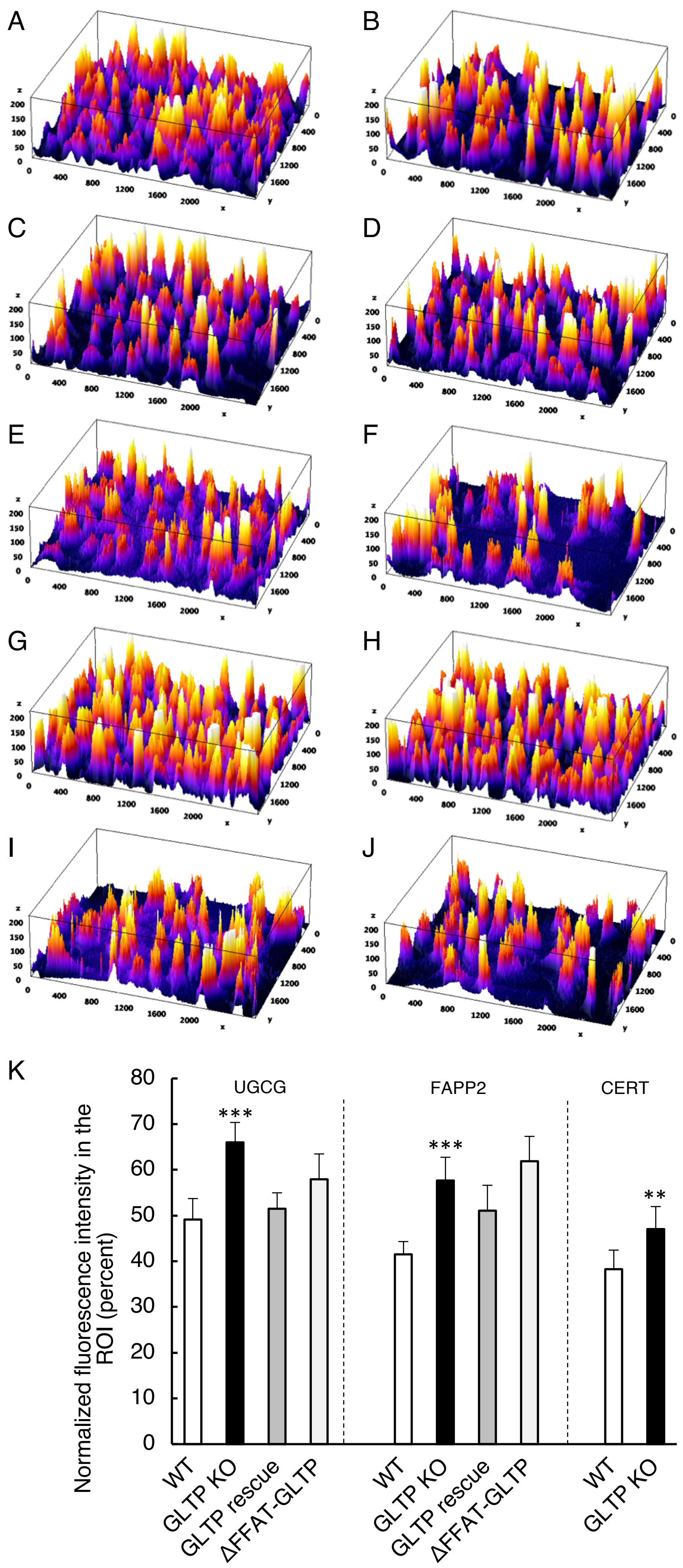
A
B
C
D
E
F
G
H
I
J
K
Normalized fluorescence intensity in the ROI (percent)
UGCG
FAPP2
CERT
***
***
**
WT
GLTP KO
GLTP rescue
ΔFFAT-GLTP
WT
GLTP KO
GLTP rescue
ΔFFAT-GLTP
WT
GLTP KO

Figure S2. **3D surface plots of HeLa cells and quantification of fluorescence intensity for UGCG, FAPP2 and CERT.** We analysed the intensity of the fluorescence in HeLa cells with the ImageJ software using the 3D interactive surface plot to further study the changes in the intracellular localization of different proteins as a function of GLTP knockout. We used ImageJ software to measure fluorescence intensity within specific regions of interest (ROI), including the nuclear, perinuclear, and ERES regions, and compared these values to the overall intensity of the entire cell body. (A) Surface plot of the fluorescence intensity for the expression of UGCG in WT HeLa cells and (B) in GLTP KO HeLa cells, (C) GLTP rescue HeLa cells and (D) ΔFFAT-GLTP expressing HeLa cells. (E) & (F) shows the fluorescence intensity for the FAPP2 protein expression in WT HeLa cells and in GLTP KO HeLa cells respectively and G) GLTP rescue HeLa cells and (H) ΔFFAT-GLTP expressing HeLa cells. (I) & (J) shows the fluorescence intensity for CERT protein expression in WT HeLa cells and in GLTP KO HeLa cells respectively. (K) The fluorescence intensity was measured in defined ROIs corresponding to ER exit sites, nuclear and perinuclear area over the fluorescence intensity of the entire cell body. Data are presented as percent of intensity for the ROI ± SEM from at least 15 cells across independent experiments. The significance in difference between the WT group was tested using a Student's t test: **$p < 0.01$, ***$p < 0.001$.

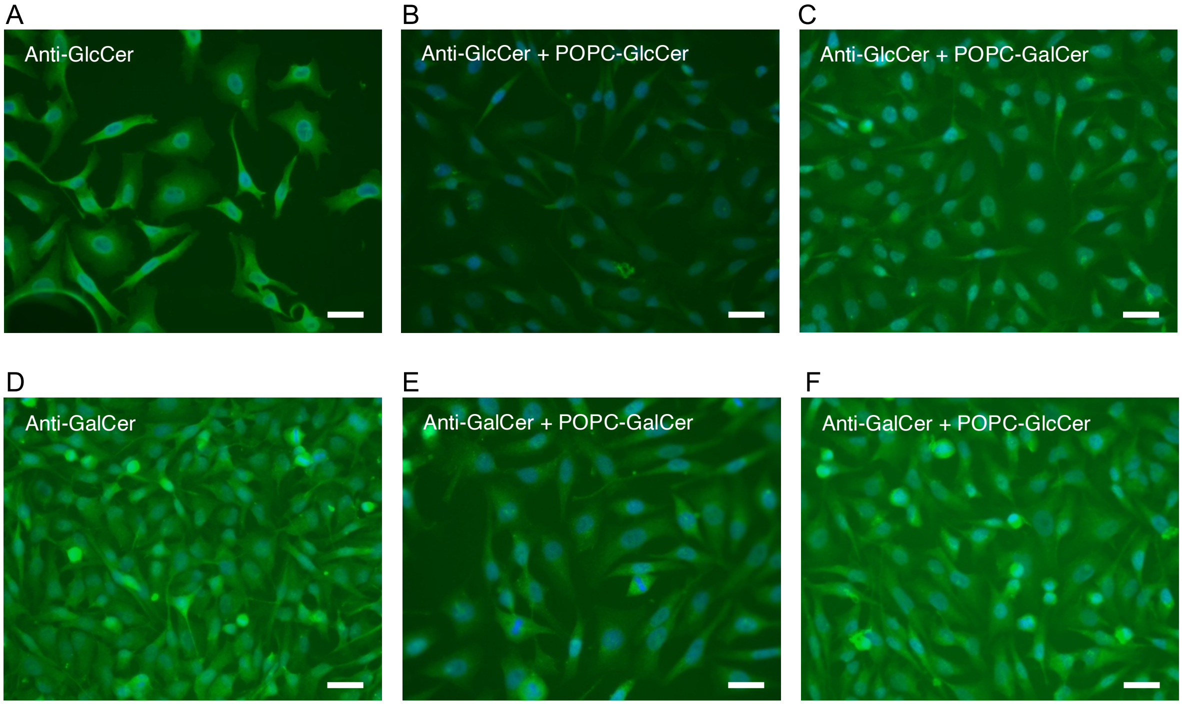

Figure S3. **Anti-GlcCer and anti-GalCer antibody verification.** The specificity of the anti-GlcCer and anti-GalCer antibodies was assayed through a competition assay using glycolipid-containing liposomes as antigens to absorb anti-GSL antibodies before using them to stain ICC samples. Anti-GSL antibodies were incubated together with liposomes composed of 90% POPC and 10% glycosphingoplipid for 60 minutes at 37C, whereafter the mixture was centrifuged in a microfuge to pellet any immune complexes. The supernatant was carefully removed and used instead of pure antibody solution to stain ICC samples. In panels B and E, in which the anti-GSL antibodies have been incubated together with their respective glycolipids (anti-GlcCer + POPC:GlcCer liposomes for panel B, anti-GalCer + POPC:GalCer liposomes for panel E) before imaging, the fluorescence intensity is lower than in panels C and F, in which the antibodies were incubated together with the non-target glycolipids (anti-GlcCer + POPC:GalCer liposomes for panel C, anti-GalCer + POPC:GlcCer liposomes for panel F). This indicates a specificity of the antibodies for their stated glycolipids, as incubation with the non-targeted glycolipid had no effect on fluorescence intensity. Panels A and C show application of untreated anti-GSL antibodies as positive controls (anti-GlcCer in panel A, anti-GalCer in panel C).
